# Complement-membrane regulatory proteins are absent from the nodes of Ranvier in the peripheral nervous system

**DOI:** 10.1186/s12974-023-02920-9

**Published:** 2023-10-24

**Authors:** Netanel Karbian, Yael Eshed-Eisenbach, Marian Zeibak, Adi Tabib, Natasha Sukhanov, Anya Vainshtein, B. Paul Morgan, Yakov Fellig, Elior Peles, Dror Mevorach

**Affiliations:** 1 Rheumatology and Rare Disease Research Center, The Wohl Institute for Translational Medicine, Hadassah-Hebrew University Medical Center and School of Medicine, Jerusalem, Israel; 2 Department of Molecular Cell Biology, Weizmann Institute of Science, Rehovot, Israel; 3 Department of Molecular Neuroscience, Weizmann Institute of Science, Rehovot, Israel; 4 Systems Immunity Research Institute, Cardiff University, Cardiff, Wales UK; 5 Department of Pathology, School of Medicine, Hadassah-Hebrew University Medical Center, Jerusalem, Israel; 6 The Institute of Rheumatology-Immunology-Allergology, The Wohl Institute for Translational Medicine, Department of Medicine, Hadassah-Hebrew University Medical Center and School of Medicine, POB 12000, 91120 Jerusalem, Israel

**Keywords:** Guillain-Barré syndrome, CD59, Peripheral nervous system, Complement, Nodes of Ranvier, Nodopathies, Myelin

## Abstract

**Background:**

Homozygous CD59-deficient patients manifest with recurrent peripheral neuropathy resembling Guillain-Barré syndrome (GBS), hemolytic anemia and recurrent strokes. Variable mutations in CD59 leading to loss of function have been described and, overall, 17/18 of patients with any mutation presented with recurrent GBS. Here we determine the localization and possible role of membrane-bound complement regulators, including CD59, in the peripheral nervous systems (PNS) of mice and humans.

**Methods:**

We examined the localization of membrane-bound complement regulators in the peripheral nerves of healthy humans and a CD59-deficient patient, as well as in wild-type (WT) and CD59a-deficient mice. Cross sections of teased sciatic nerves and myelinating dorsal root ganglia (DRG) neuron/Schwann cell cultures were examined by confocal and electron microscopy.

**Results:**

We demonstrate that CD59a-deficient mice display normal peripheral nerve morphology but develop myelin abnormalities in older age. They normally express myelin protein zero (P0), ankyrin G (AnkG), Caspr, dystroglycan, and neurofascin. Immunolabeling of WT nerves using antibodies to CD59 and myelin basic protein (MBP), P0, and AnkG revealed that CD59 was localized along the internode but was absent from the nodes of Ranvier. CD59 was also detected in blood vessels within the nerve. Finally, we show that the nodes of Ranvier lack other complement-membrane regulatory proteins, including CD46, CD55, CD35, and CR1-related gene-y (Crry), rendering this area highly exposed to complement attack.

**Conclusion:**

The Nodes of Ranvier lack CD59 and are hence not protected from complement terminal attack. The myelin unit in human PNS is protected by CD59 and CD55, but not by CD46 or CD35. This renders the nodes and myelin in the PNS vulnerable to complement attack and demyelination in autoinflammatory Guillain-Barré syndrome, as seen in CD59 deficiency.

**Supplementary Information:**

The online version contains supplementary material available at 10.1186/s12974-023-02920-9.

## Background

Guillain-Barré syndrome (GBS), including its variants, is currently the most frequent cause of acute flaccid paralysis worldwide and constitutes a serious neurological emergency. The disease encompasses a group of peripheral nerve disorders, each distinguished by the distribution of weakness in the limbs or cranial nerve-innervated muscles and underlying pathophysiology. There is substantial evidence to support an autoimmune cause with autoantibodies for the classical syndrome [1] and an autoinflammatory pattern without autoantibodies in congenital mutated CD59 [2–4]. In both conditions, complement activation has been suggested to have a crucial role in pathogenesis, although it is not known why patients with GBS or those with CD59 deficiency are susceptible to complement attack [1–4].

Primary congenital CD59 deficiency manifested by recurrent GBS in humans is a germline mutation described in 18 individuals and characterized by recurrent GBS (17/18), hemolytic anemia (14/16), and recurrent strokes (8/18) [3–17].

The membrane attack complex (MAC) inhibitory function of CD59 is related to its capacity to bind C8 within C5b-8 and block recruitment of C9, which is essential for MAC formation. This accounts for the observed impact on lytic sensitivity [18, 19]. We have recently shown that the four CD59 mutations described so far have similar clinical manifestations despite differences in their mutant proteins [2]. Frameshift mutants p.Asp24Valfs* and p.Ala16Alafs* generate truncated proteins with a distinct c-terminal sequence, common to both mutants, that are not present on the plasma membrane. In contrast, missense mutants p. Cys64Tyr and p.Asp24Val do reach the cell surface. Despite this difference, all four mutants produce nonfunctional CD59 and are thus unable to protect the host from MAC attack. As judged by clinical manifestations, another two mutations, the Ser83Ter (terminal) nonsense [8] and Tyr4Asp missense mutations [9], produce nonfunctional CD59.

How would nonfunctioning mutated CD59 contribute to the appearance of both autoimmune (classical sporadic) and autoinflammatory (congenital, recurrent) GBS? In the current study, we use biopsies from healthy and CD59-deficient humans and from wild-type (WT) and CD59a-deficient mice. We evaluated cross sections and teased sciatic nerve fibers and myelinating dorsal root ganglia (DRG) neuron/Schwann cell cultures for CD59 localization using a variety of antibodies to study the exact anatomical localization of CD59 and other membrane-bound complement with the aim of understanding their physiological and pathophysiological roles.

## Methods

### Ethical approval

Human (HMO-12-0390; HMO-18-0379) and animal (MD-17-14937-4; MD-12-13432-1) ethical approvals were issued by the committees of Hadassah Medical Center and Hebrew University Faculty of Medicine, respectively.

### Immunofluorescence labeling of complement regulators in murine peripheral nerve

Mouse sciatic nerves were excised and fixed for 30 min in 4% paraformaldehyde. For cross-sections and longitudinal sections, all mouse sciatic nerves were cryoprotected overnight in 30% sucrose and then cut into 10 μm-thick sections. Teased sciatic nerves, cross sections, and longitudinal sections were immunostained as described previously [20]. Briefly, samples were permeabilized by methanol and blocked for 1 h with 0.5% Triton-X 100 in PBS and 5% normal goat serum, then incubated overnight at 4 °C with different primary antibodies diluted in 0.1% Triton-X 100 in PBS blocking solution, extensively washed, and incubated for 45 min at room temperature (Rt) with secondary antibodies. Finally, slides were mounted with elvanol and analyzed.

### Immunohistochemistry (IHC) labeling of complement regulators in murine peripheral nerve

This labeling was also performed with the tyramide signal amplification (TSA) method in teased fibers and longitudinal sections. Endogenous peroxidase was quenched by incubation for 30 min in 0.25% H_2_O_2_/methanol. Sections were washed in PBS and incubated for 30 min with blocking buffer (TSA Fluorescence System Kit, PerkinElmer, Waltham, MA, USA). They were then incubated for 30 min with primary antibodies, either with 3 µg/ml rat antibody against mouse DAF/CD55 (Cardiff University, 2C6) or with rabbit polyclonal antibody against mouse Complement receptor type-1 related gene Y (Crry (, Biorbyt, Cambridge, UK). The treated sections were subsequently washed with TNT buffer (0.1 M Tris, pH 7.5, 0.15 M NaCl, 0.05%, Tween-20) and incubated for 30 min with 3 µg/ml horseradish peroxidase (HRP)-conjugated mouse anti-rat/rabbit IgG (Jackson Laboratories, Bar Harbor, ME, USA). After a second wash with TNT buffer, the sections were stained for 6 min at 20 °C with tyramide-red (PerkinElmer). Finally, slides were mounted with elvanol.

For murine sciatic nerve and teased cross- and longitudinal sections (including those prepared with the TSA method), we used rabbit polyclonal antibody (pAb) against mouse CD59 (Cardiff University), rat monoclonal antibody (mAb) against mouse CD59 (Cardiff University), chicken pAb against mouse myelin protein zero (P0, AVES-2B Scientific, Oxfordshire, UK), mouse mAb against mouse ankyrinG (AnkG, NeuroMab, Davis, CA, USA), rat mAb against mouse myelin basic protein (MBP) (Millipore, Burlington, MA, USA), rabbit mAb against mouse Caspr (Weizmann Institute [21]), rat mAb against mouse DAF/CD55 (Cardiff University, 2C6), rabbit pAb against mouse Crry (Biorbyt, Cambridge, UK), rabbit pAb against mouse CD46 (Abcam, Cambridge, UK), mouse mAb against mouse beta dystroglycan (Novocastra, Newcastle on Tyre, UK), mouse mAb against mouse myelin-associated glycoprotein (MAG) (Chemicon International, Temecula, CA, USA), chicken pAb against mouse neurofascin (Nfasc, R&D, Minneapolis, MN, USA), rabbit pAb against mouse CD31 (Abcam, Cambridge, UK), and rat mAb against mouse CD31 (Abcam, Cambridge, UK). Fluorophore-coupled secondary antibodies included Cy3-coupled anti-rabbit, anti-mouse, and anti-rat IgG; 488-coupled anti-rabbit, anti-mouse, and anti-rat IgG; and Cy5-coupled anti-chicken IgG (Jackson Laboratories).

For mouse DRG culture we used rabbit pAb against mouse CD59 (Cardiff University), rat mAb against mouse MBP (Millipore, Burlington, MA, USA), and chicken mAb against mouse Nfasc (R&D, Minneapolis, MN, USA). Fluorophore-coupled secondary antibodies included Cy3-coupled anti-rat IgG, Cy5-coupled anti-chicken IgG, and 488-coupled anti-rabbit IgG (Jackson Laboratories).

### Immunofluorescence staining for human sural nerve

Paraffin-embedded sections from human sural nerve biopsies were also stained by immunofluorescence labeling using mouse mAb against human CD59, rabbit pAb against human CD31, and mAb against human MBP.

All purified antibodies were used in concentrations of 1–5µg/ml according to manufacturer recommendations. Serum antibodies were used in dilutions of 1 × 10^–3^–1 × 10^–4^.

Fluorescence images were obtained using a confocal microscope (LSM700, Carl Zeiss, Oberkochen, Germany) fitted with an ORCA-ER CCD camera (Hamamatsu, Hamamatsu, Japan). Images were acquired and processed using the Zen2012 (Carl Zeiss) and PhotoShop software (Adobe, San Jose, CA, USA).

### Immunohistochemistry labeling of complement regulators in human peripheral nerve

Paraffin-embedded sections from human sural nerve biopsies were stained with hematoxylin and eosin (H&E), Luxol fast blue-periodic acid Schiff (LFB-PAS), Bielschowsky, Gomori-trichrome (GTC), and Congo-red according to the standard procedures. Paraffin-embedded sections of human sural biopsies were stained using IHC methods for mouse mAb against human CD59 (BIO-RAD, Hercules, CA, USA) at 1:400 dilutions, rabbit mAb against human CD55 antibody (Abcam, CB, UK) at 1:400 dilution, rabbit mAb against human CD46 (Abcam, CB, UK) at 1:1000 dilution, mouse mAb against human CD35 (Abcam, CB, UK) at 1:100 dilution, rabbit pAb against human MBP (Zymed, Waltham, MA, USA), mouse mAb against human neurofilament (NF) (Dako, Glostrup South, Denmark), and rabbit pAb against human S-100 (Dako, Glostrup South, Denmark). Universal secondary HRP antibody (UltraView Universal HRP Multimer) was used for all staining (Ventana, Tucson, AZ, USA). All IHC was performed using an automated stainer (Ventana Benchmark-Ultra, Roche Diagnostics, Indianapolis, IN, USA) according to standard procedures and protocols. Staining peripheral nerve for autoantibodies was done using patients’ sera at a dilution of 1:100 or IVIg control at 1:10,000. mAb against mouse βIV spectrin 1:600 and mAb against mouse neurofascin, 1:300, were also used for this set of stains.

Images were obtained using a Nikon Ti automated inverted microscope fitted with a Nikon DS-Fi1 color CCD camera and by Andor Dragonfly 200, a spinning disc confocal microscope. Images were acquired and processed using NIS-Elements image acquisition software (Nikon) or Fusion software (Andor).

### Myelinating dorsal root ganglia (DRG) cultures

DRG cultures were prepared from mouse embryos on day 13.5 of gestation as previously described [22]. DRGs were dissociated and plated at a density of 4 × 10^4^ per 13 mm slide and coated with matrigel and poly-l-lysine. Cultures were grown for 2 days in neurobasal medium (Gibco) supplemented with B-27 (Gibco), glutamax (Gibco), penicillin /streptomycin, and 50 ng/ml of nerve growth factor (NGF, Alomone Labs). Cultures were then grown for 4–5 additional days in BN medium containing Basal Medium Eagle (Sigma), ITS supplement (Sigma), glutamax (Gibco), 0.2% BSA, 4 mg/ml d-glucose, 50 ng/ml NGF, and antibiotics. To induce myelination, cultures were grown in BNC, namely a BN medium supplemented with 15% heat-inactivated fetal calf serum (Gibco, replacing the BSA) and 50 μg/ml l-ascorbic acid. DRG were fixed in 4% PFA on day 17.

### Electron microscopy (EM)

Adult WT and CD59a knock-out (KO) mice were anesthetized using a lethal dose of ketamine/xylazine (1:10) injected intraperitoneally. Sciatic nerves were fixed for 30 min in situ with a freshly prepared fixative containing 4% paraformaldehyde, 2.5% glutaraldehyde, 0.13 M NaH_4_PO_4_, and 0.11 M NaOH pH 7.4. Nerves were subsequently dissected out and cleaned from surrounding tissue, and further fixed with the above fixative overnight at room temperature while shaking. Fixed sciatic nerves were analyzed using a transmission electron microscope (CM-12, Eagle 2K × 2K; FEI, Philips, Amsterdam, The Netherlands). Semi-thin sections were stained with toluidine blue (TB) for further analysis as described previously [23].

### Statistical analysis

The unpaired two-tailed Student’s t-test was used to compare axoplasmatic organelles from WT and CD59a-deficient mice.

## Results

### Localization of CD59 in murine sciatic nerve and myelinated cultures

**Fig. 1:**
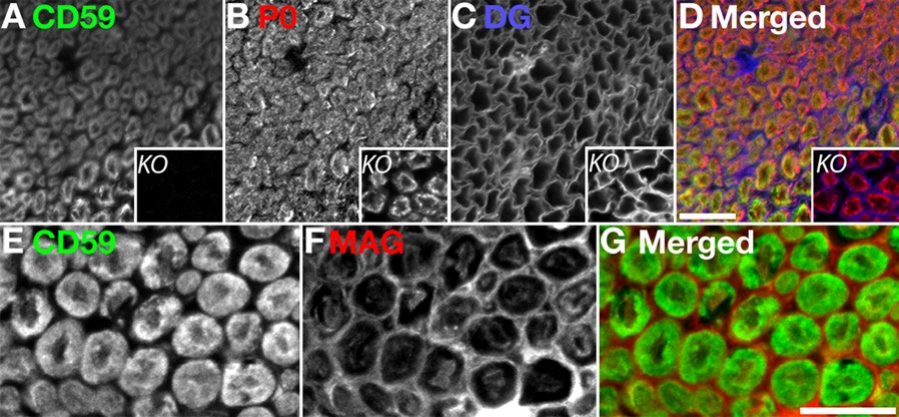
Localization of CD59 in peripheral nerve and murine sciatic nerve cross-sections. **A**–**D** CD59 (green), P0 (red) and beta dystroglycan (DG, blue) staining of murine wild-type (WT) and knock-out (KO) cross section fibers (**A**-**D** small squares). **E**–**G** CD59 (green) and myelin-associated glycoprotein (MAG, red) staining of fibers from WT mice. Staining shows that CD59 and P0, which represent compact myelin, have identical patterns in WT mice. Sections were taken from 4-month-old mice. Immunolabeling was performed with methanol and 0.1% triton. Scale bars 20 μm

**Fig. 2:**
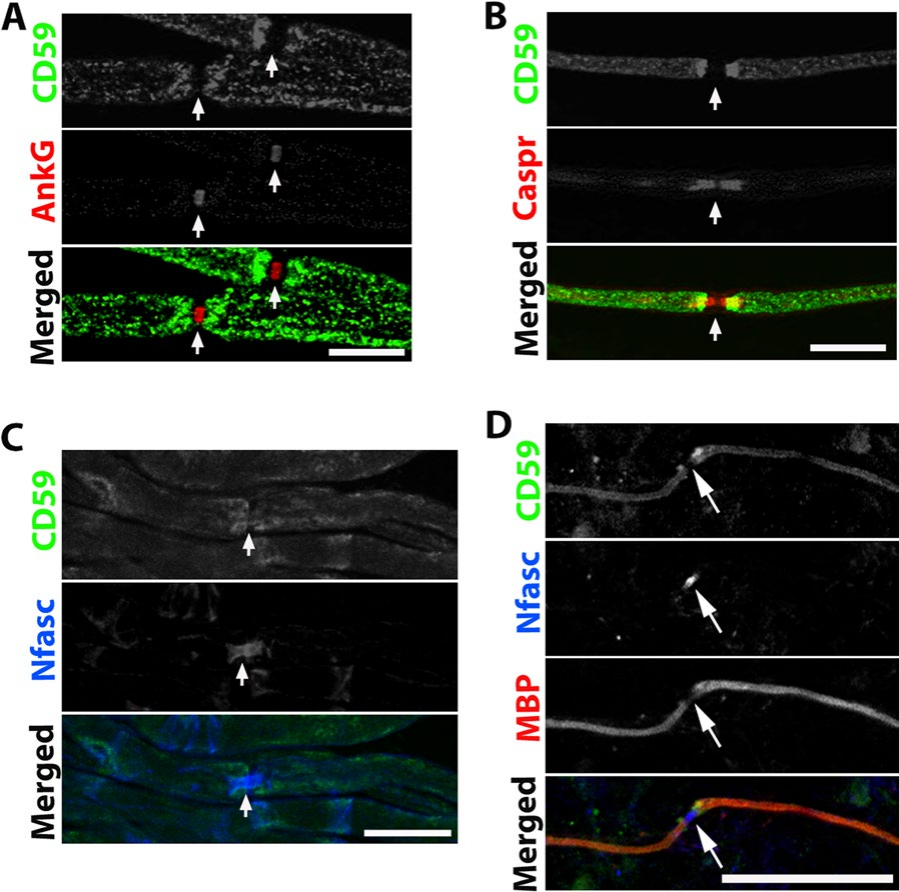
Localization of CD59 in murine teased nerve and myelinated cultures. **A** CD59 (green) and ankyrin-G (AnkG, red) staining of WT teased fibers. **B** CD59 (green) and Caspr (red) staining of WT teased fibers. **C** CD59 (green) and neurofascin (Nfasc, blue) staining of WT teased fibers. **D** CD59 (green), myelin basic protein (MBP, red), and neurofascin (Nfasc, blue) staining of WT ICR DRG. CD59 is co-localized with MBP and localized in the internodes and paranodes. There is no CD59 localization in the nodes of Ranvier. **A**–**C** Sections taken from a 4-month-old mouse. Immunolabeling procedure handled with methanol and 0.1% triton. Scale bars 20 μm and in **D** 50 μm

To evaluate the localization of CD59 in the peripheral nervous system (PNS), we labeled cross sections of sciatic nerves from both WT and CD59a KO mice with antibodies to CD59 together with antibodies to dystroglycan and P0 which mark the outer aspect of the myelin unit facing the basal lamina and the compact myelin, respectively. This immunolabeling revealed that CD59 is present in compact myelin and merges with P0 in WT nerves (Fig. 1A–D). As expected, CD59 was not detected in KO sciatic nerves (Fig. 1A–D, small squares), confirming the antibody’s specificity. Immunolabeling of anti-CD59 together with anti-MAG, a non-compact myelin protein that is present in the outer (abaxonal) and inner (adaxonals) Schwann cell membranes, showed nonoverlapping signals, confirming that CD59 was indeed localized in an area resembling the compact myelin (Fig. 1E–G). This was further supported by immunolabeling of WT nerves using antibodies to CD59 and AnkG, which revealed that CD59 was localized along the internodal areas but was absent from the nodes of Ranvier (Fig. 2A). Additional immunolabeling of WT nerves using antibodies to CD59, and other markers of the nodes of Ranvier [24], including Caspr and Nfasc, showed that CD59 was also present in non-compact myelin areas like the paranodal loops and Schmidt Lanterman incisure (SLI) (Fig. 2B, C). Caspr is present in the paranodal loop domain while Nfascis present in paranodal loops and SLI).

To further characterize the localization of CD59 in peripheral nerve myelin, and in particular its absence in the nodes of Ranvier, we immunolabeled myelinated Schwann cells/DRG neuron cultures using antibodies to MBP and P0 together with CD59. These experiments revealed that CD59 was absent from the nodes of Ranvier and colocalized with MBP (Fig. 2D) and P0 (Additional file 1: Fig. S1) in WT cultures.

### Localization of CD59 in human sural nerve

**Fig. 3:**
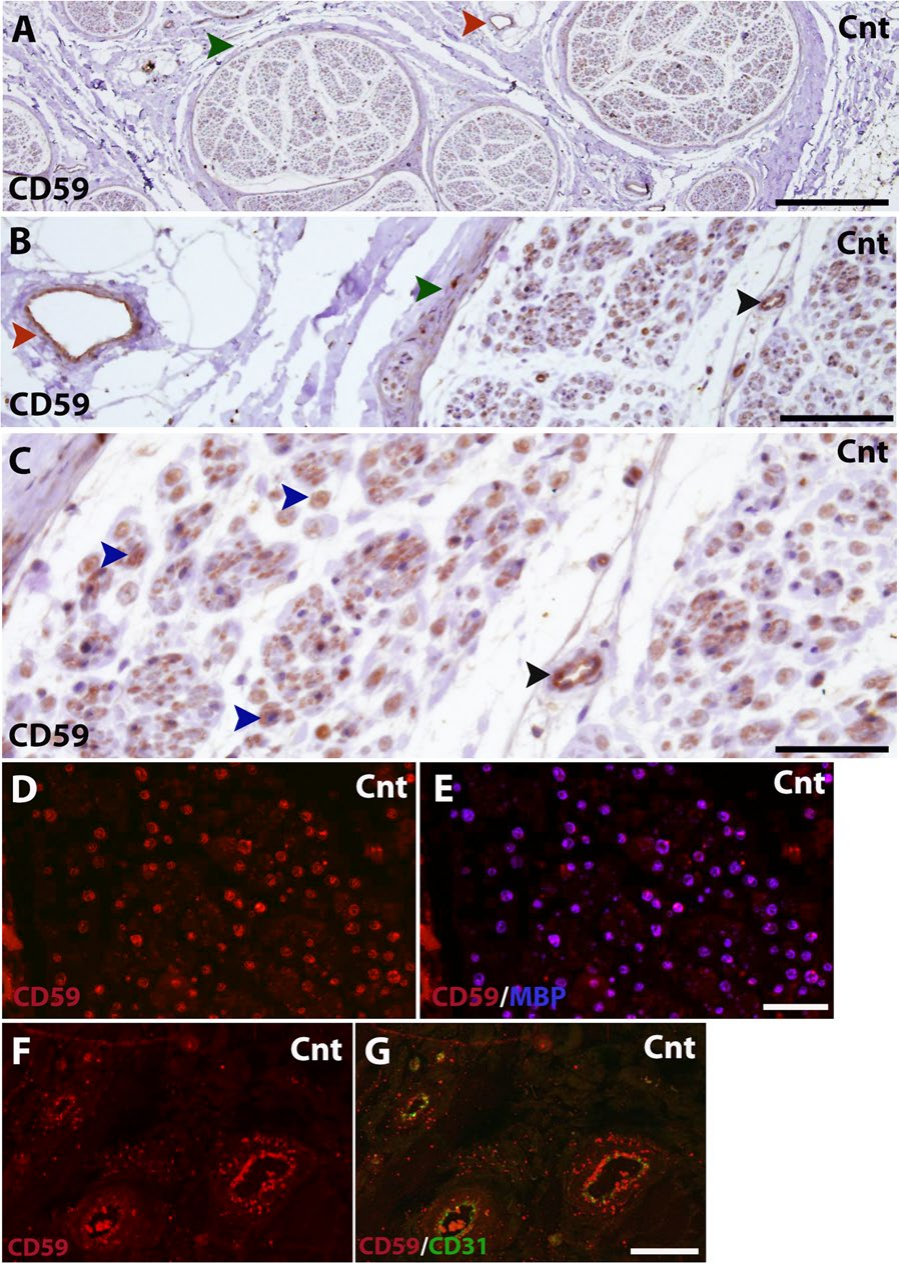
Localization of CD59 in human sural nerve. **A**–**C** Immunohistochemical staining for CD59 (DAB, red) in sural nerves of a healthy control. Healthy control staining shows compact myelin localization (**C**, blue arrow heads). CD59 was also detected in endothelial blood vessels of the epineurium (**A** and **B**, red arrow heads) and endoneurium (**B** and **C**, black arrow heads), as well as the perineurium (**A** and **B**, green arrow heads). **D**, **E** Immunofluorescence staining for CD59 (red) and MBP (blue). **F**, **G** Immunofluorescence staining for CD59 (red) and CD31 (green). Scale bars: **A** 500 μm, **B** 100 μm, **C**–**G** 50 μm. Magnifications: **A** ×4, **B**, **D**–**G** and ×20, **C** ×40. **A**–**C** 1:400 dilution

To test whether the observations made in mouse nerves apply to humans, we first tested the localization of CD59 in paraffin-embedded cross sections of healthy human sural nerve. As seen in the results obtained in mice, immunohistochemical staining revealed CD59 localization in the area of myelinated nerve fibers (Fig. 3C, blue arrow heads). CD59 was also detected in the endothelial blood vessels of the epineurium and endoneurium (Fig. 3A–C, red arrow heads for epineurium, black arrow heads for endoneurium), and in the perineurium (Fig. 3A, B, green arrow heads). Immunolabeling of anti-CD59 together with anti-MBP showed overlapping signals, confirming that CD59 was indeed localized in compact myelin (Fig. 3D, E). Immunolabeling of anti-CD59 together with anti-CD31 also showed overlapping signals, confirming that CD59 was indeed localized in endothelial blood vessels (Fig. 3F, G).

### CD59 is not required for murine PNS myelination, and its absence leads to abnormal myelination only at older age

**Fig. 4:**
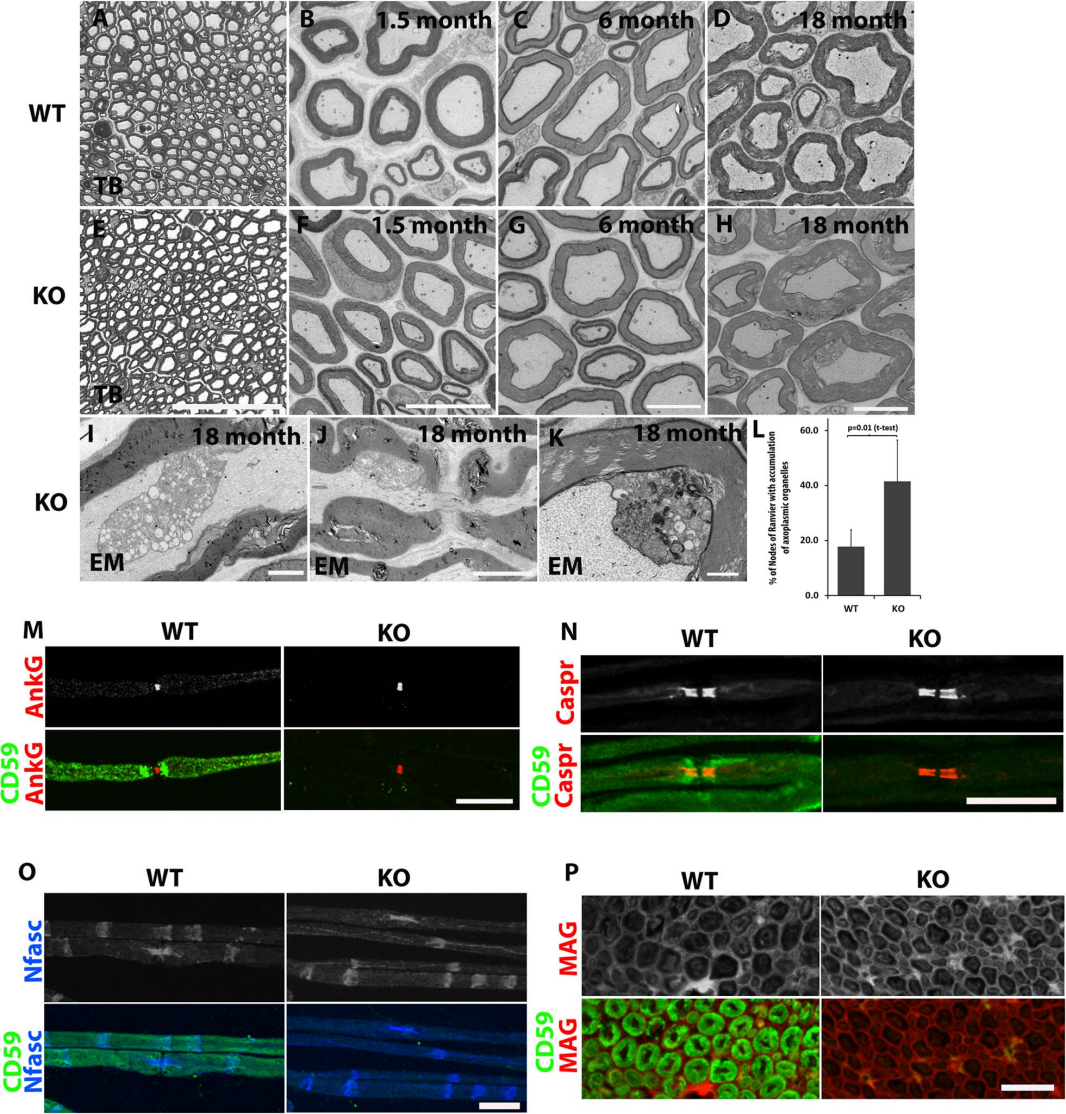
Electron microscopy (EM) and immunofluorescence labeling of murine sciatic nerve. EM pictures of cross sections from WT (**A**–**D** (and CD59a-deficient (**E**–**H**) murine sciatic nerve at different ages. **A** and **E **are low-resolution toluidine blue pictures from a 6-month-old mouse. Scale bars: **A** and **E** 50 μm, **B** and **F** 10 μm, **C**, **D**, **G** and **H** 5 μm. **I**–**L** EM (higher magnification) showing accumulation of axoplasmatic organelles at an older age (18 months) in longitudinal sections from the paranodal regions (**I**, **J**, scale bars: **I** 2 μm, **J** 5 μm), and mitochondria and dense bodies in cross sections at regions with of noncompact myelin (**K**, scale bar = 500 nm; **I**–**K** are from CD59 KO mice). WT vs CD59 KO mice, comparison of the percentage of nodes of Ranvier with accumulation of axoplasmic organelles is shown in **L** (WT: n = 5, 82 nodes. KO: n = 5, 117 nodes; (p = 0.01, unpaired two-tailed student’s t-tests). **M**–**P** Immunofluorescence labeling of WT (left panels) and CD59 KO (right panels) teased fibers show normal node structure by CD59 (green) and AnkG (**M,** red), Caspr (**N**, red), neurofascin (**O**, Nfasc, blue), and myelin-associated glycoprotein (**P**, MAG, red). Sections taken from a 4-month-old mouse. Immunolabeling procedure handled with methanol and 0.1% triton. Scale bars 20 μm

To examine whether genetic deletion of CD59 affected myelin morphology, we compared cross sections of sciatic nerves obtained from WT and KO animals by EM and TB staining. We found no significant morphological differences between the WT and KO nerves (Fig. 4A, E). Similar results were obtained from 1.5 and 6-month-old animals (Fig. 4B, C, F, G). EM analysis of 18-month-old animals revealed abnormalities in both genotypes (Fig. 4D, H–L), including the appearance of conspicuous axoplasmatic organelles that were often present in the paranodal region (Fig. 4I, J). These axoplasmatic organelles were more frequently detected in KO compared to WT mice, with an average of 41.5 in KO mice compared to 17.8 in WT mice (n = 5, p = 0.01) (Fig. 4L). In addition, in the KO nerves we detected areas that contained mitochondria and dense bodies (Fig. 4K), and some neurofilaments that had lost their normal orientation and formed disorganized bundles (Additional file 2: Fig. S2). These results may indicate that CD59a KO nerves are more sensitive to nerve damage and degeneration, suggesting a protective role for CD59 in the PNS.

To further establish that genetic deletion of CD59 did not affect myelin morphology, we compared immunolabeling of sciatic nerve stains obtained from 4-month-old WT and KO animals using antibodies to CD59 and AnkG (Fig. 4M), Caspr (Fig. 4N), Nfasc (Fig. 4O), and MAG (Fig. 4P). There was no significant difference in the intensity or localization of these molecular markers between WT and KO nerves, indicating that the molecular organization of the myelin unit is preserved in the absence of CD59 (Fig. 4M–P).

### Localization of CD59 in the peripheral nerves of CD59-deficient patients

**Fig. 5:**
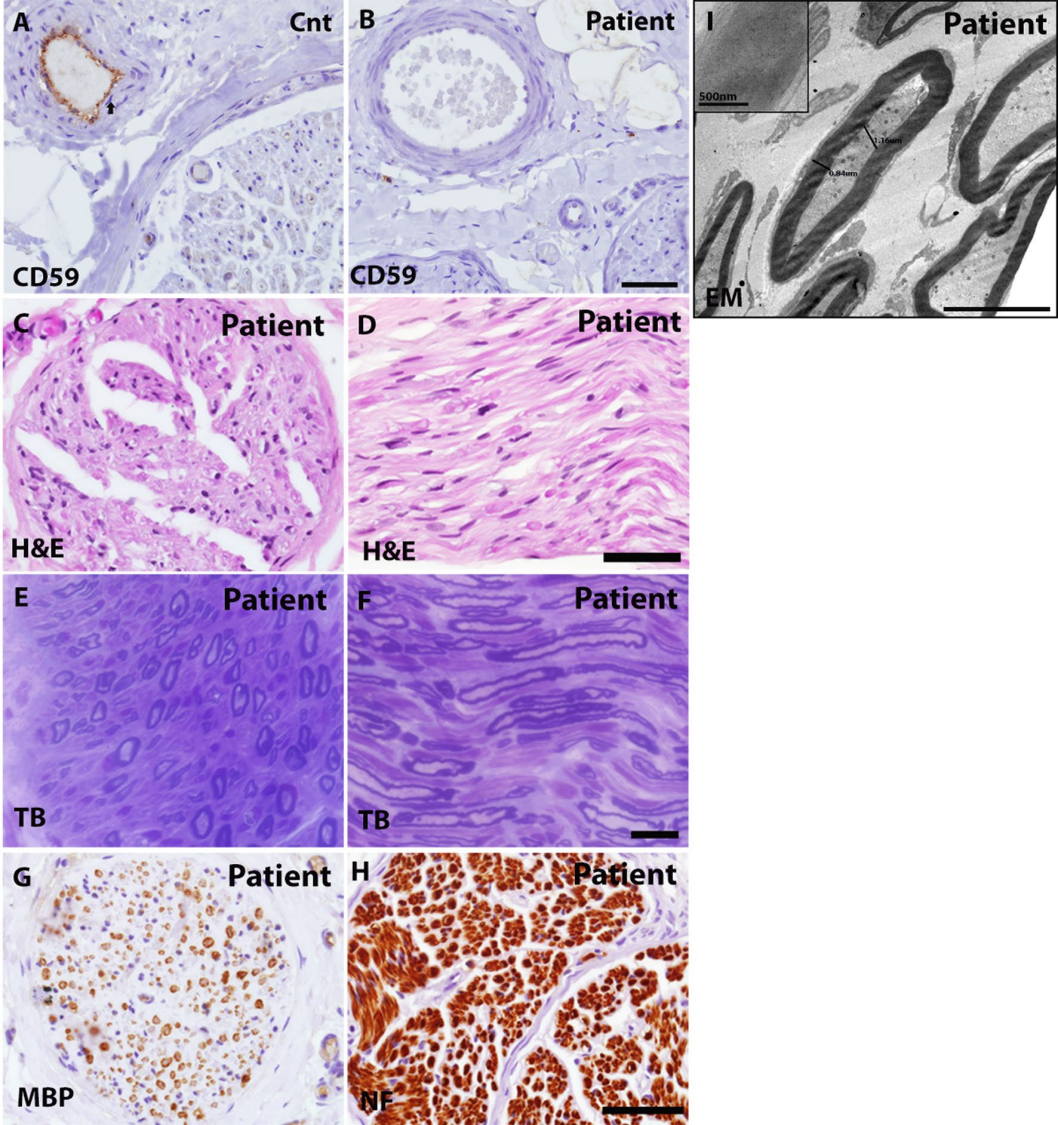
CD59 patient sural nerve biopsy. **A**, **B** Immunohistochemical staining of CD59 (DAB, red) from a healthy control subject (**A**) and a CD59 patient (**B**). CD59-deficient patient staining of CD59 was completely absent in comparison to a healthy control subject where CD59 appears to localize in compact myelin. Scale bars = 50 μm, magnifications × 40, 1:400 dilution. **C**–**H** Hematoxylin and eosin (H&E) (**C **and** D**), epoxy-resin embedded semi-thin sections stained with toluidine-blue (**E** and **F**), immunohistochemical staining for MBP (**G**) and neurofilament (NF) (**H**). Staining displayed normal nerve fiber density without evidence of active axonal or myelin damage. Scale bars: **C**, **D**, **G** and **H** 50 μm; **E** and **F** 20 μm; magnifications: **C**, **D**, **G** and **H** ×40; **E** and **F** ×60. **I** Electron microscopy image, tissue from a CD59-deficient patient showing thin myelin sheaths relative to axonal diameter with a relatively high g-ratio, suggestive of remyelination. Scale bar 5 μm. small squares, myelin sheath lamellar structure. Scale bar 500 nm

To test whether the observations made in mouse nerves apply to humans, we also examined paraffin-embedded sections of sural nerve biopsied from a CD59-deficient patient between GBS episodes. CD59 protein was not detected with IHC staining in patient cross sections but was seen in cross sections from healthy control subjects (Fig. 5A, B). Paraffin-embedded sections from a CD59-deficient patient were stained with H&E (Fig. 5C, D), LFB-PAS, Bielschowsky, GTC, and Congo-red (data not shown). Epoxy resin-embedded semi-thin sections stained with TB (Fig. 5E, F), IHC staining for MBP (Fig. 5G), NF (Fig. 5H), and S-100 protein (data not shown) displayed normal nerve fiber density. Nerve fibers appeared normal, without evidence of active axonal or myelin damage. There was no apparent excess of endoneurial cells or collagen. Neither inflammatory infiltrate nor amyloid were identified. EM (Fig. 5I) showed occasional thin myelin sheaths relative to axonal diameter with a relatively high g ratio (inner axonal diameter: total diameter) of up to 0.8 μm compared to 0.5–0.7 μm seen in healthy axons [25, 26], which is suggestive of segmental demyelination/remyelination. The g-ratio axonal diameter, including the myelin sheath, was calculated using EM. The myelin sheath lamellar structure appeared to be normal. No macrophages or other signs of active axonal or myelin damage were identified. Axons and Schwann cells were unremarkable. These findings suggest that in CD59-deficient patients there is a process of demyelination followed by remyelination, leading to a relatively thinner myelin layer.

### No autoantibodies to peripheral nerves are found in the sera of children with CD59 deficiency

We examined autoantibodies that classically localize in GBS in the serum of four children with CD59 deficiency. No anti-Ganglioside D1b (GD1b), nor anti-Ganglioside-monosialic acid 1 (GM1), were found in their serum during 1–7 years following diagnosis (data not shown). We also exposed teased mouse peripheral nerve fibers to their serum, collected up to 5 years after the diagnosis, with no detection of autoantibodies (data not shown).

### Differentially localized complement membrane regulatory proteins in peripheral nerve and endothelial blood vessels

Membrane-bound complement regulatory proteins include CD59, CD55, CD46, and CD35 (complement receptor 1, CR1) in humans, and CD59, CD55, CD46, and Crry in mice [27–29].

As shown in Figs. 1, 2, CD59 was absent from the nodes of Ranvier and co-localized with MBP and P0 in WT mice. To determine whether CD59 is localized in endothelial blood vessels in mice, we labeled longitudinal sections of murine sciatic nerve with antibodies to CD59, Nfasc, and CD31 as an endothelial blood vessel marker. Immune-labeling revealed CD59 localization in internodes corresponding to compact myelin but there was no CD59 localization in blood vessels (Additional file 3: Fig. S3 A, B). In humans, however, and as discussed earlier, CD59 was also detected in endothelial blood vessels of the epineurium, endoneurium, and perineurium (Fig. 3G–C).

**Fig. 6:**
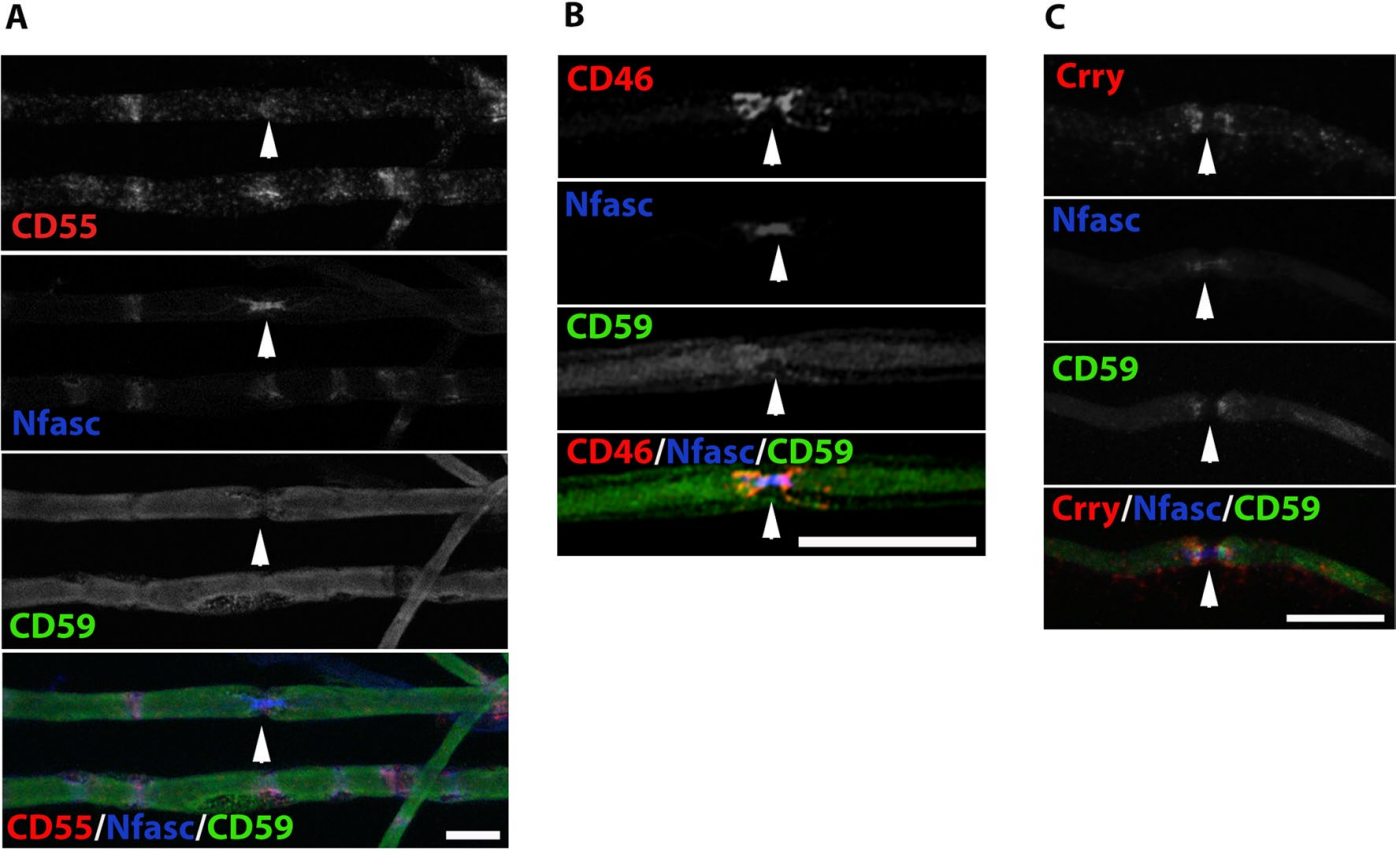
Localization of complement membrane regulatory proteins in murine teased fibers of sciatic nerve. Staining of WT teased fibers. CD55 (red), neurofascin (Nfasc, blue) and CD59 (green) (**A**), CD46 (red), neurofascin (Nfasc, blue) and CD59 (green) (**B**), Crry (red), neurofascin (Nfasc, blue) and CD59 (green) (**C**). Sections taken from a 4-month-old mouse**.** Immunolabeling procedure performed with the TSA method (**A**, **C**) and methanol and 0.1% triton (**B**). Scale bars 20 μm. CD55 staining was localized in Schmidt Lanterman incisures. CD46 staining was localized in the paranodes-juxtaparanodes, and Crry staining was localized in the paranodes and the internodes corresponding to compact myelin. White arrowheads indicate the nodes of Ranvier

**Fig. 7:**
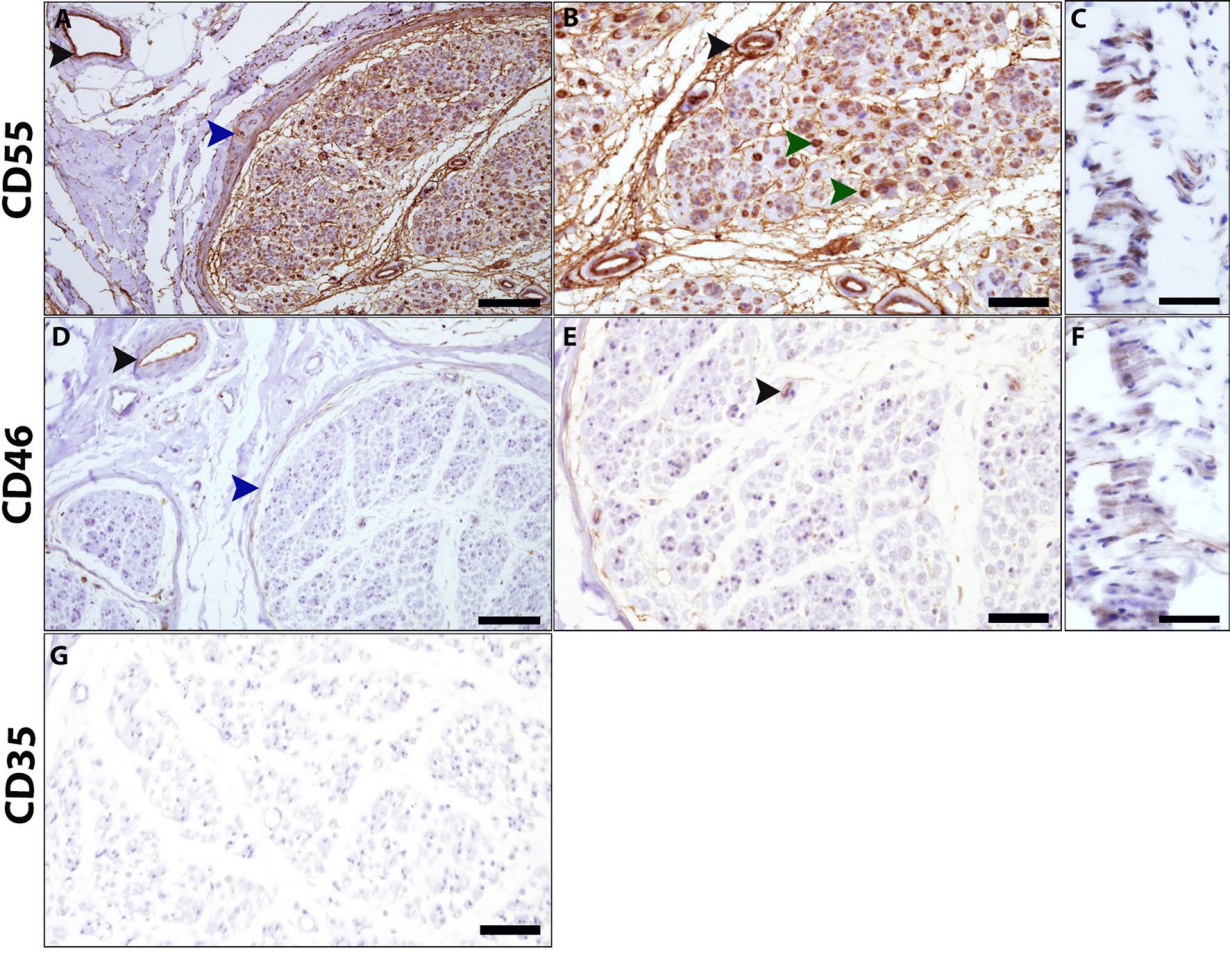
Complement membrane regulatory proteins in human peripheral sural nerve.** A**–**G.** Human sural nerve from a healthy control. Cross and longitudinal section immunohistochemical staining for CD55 (DAB, red) (**A**–**C**), CD46 (DAB, red) (**D**–**F**) and CD35 (**G**). CD55 staining corresponds to compact myelin (**B**, green arrow heads) and endothelial blood vessels of the epineurium (**A**, black arrowhead) and endoneurium (**B**, black arrowhead), as well as the perineurium (**A**, blue arrowhead). CD46 was detected in endothelial blood vessels of the epineurium (**D**, black arrowhead) and endoneurium (**E**, black arrowhead), as well as the perineurium (**D**, blue arrowhead). CD35 was not detected anywhere. Scale bars: **A**, **D** 100 μm; **B**, **C**, **E**–**G** 50 μm. Magnifications: **A**, **D** ×20; **B**, **C**, **E**–**G** ×40

Interestingly, CD55 was not present in compact myelin in mouse peripheral nerve teased fibers and was expressed in SLI (Fig. 6A and Additional file 4: Fig. S4A). This observation was confirmed in murine longitudinal sections (Additional file 4: Fig. S4B). No CD55 was detected in the endothelial blood vessels, and no colocalization with antibodies to CD31 was seen (Additional file 4: Fig. S4B). However, normal human IHC staining cross sections revealed that CD55 was localized in a manner similar to CD59, in compact myelin and in the endothelial blood vessels of the epineurium, endoneurium, and perineurium (Fig. 7A, B). In longitudinal human sural nerve sections, CD55 was localized in areas that correspond to exon fibers and compact myelin (Fig. 7C).

Taken together, this suggests that CD55 is localized in myelin and blood vessels in human peripheral nerves, whereas in mice, CD59 localization is seen in compact myelin but CD55 is localized in SLI of the noncompact myelin compartment.

As shown in Fig. 6, CD46 was localized in the paranodal and juxtaparanodal domains and not in the nodes or compact myelin in murine peripheral nerve teased fibers (Fig. 6B). Immune-labeling for mouse CD46, Nfasc, and CD31 as an endothelial blood vessel marker in murine longitudinal sections, confirmed that CD46 was localized in the paranodal-juxtaparanodal area, and was also weakly localized in the endothelium of blood vessels (Additional file 5: Fig. S5 A-B).

In human sural nerve cross sections, CD46 was localized in endothelial blood vessels of the epineurium and endoneurium, and to a lesser extent in the perineurium (Fig. 7D–F). These results suggest that CD46 is localized in blood vessels of the peripheral nerve in both humans and mice, and in the paranodal region of murine peripheral nerves.

CD35 (CR1) was not detected in the human peripheral nervous system (Fig. 7G).

An additional complement regulator, Crry, an inhibitor of complement activation, is localized in rodent, but not human cell membranes, and is a functional and structural analog of human CD55/DAF and CD46/MCP [30]. We therefore stained for Crry,and found that it is localized in the paranodal areas and in the internodes, corresponding to compact myelin (Fig. 6C and Additional file 6: Fig. S6A-B). Thus, as shown in Figs. 1, 2, 3 and 6, 7, complement membrane-regulatory proteins are differentially localized in the PNS in mice and humans. In compact myelin, CD59 and CD55 are expressed in humans, whereas CD59 and Crry are localized in mice. In endothelial blood vessels, CD59, CD55, and CD46 are localized in humans, whereas only CD46 is localized in mice. These observations may explain human clinical observations associated with CD55 and CD59 deficiencies. In patients with CD59 deficiency, CD59 is not present in the nodes of Ranvier (classical sporadic GBS), and as it is normally abundant in compact myelin (congenital recurrent GBS), its absence leads to GBS [2, 3] whereas CD55 deficiency does not [31, 32]. In mice, however, lack of CD59 does not lead to GBS following viral infection (Karbian and Mevorach, unpublished data) as Crry may prevent complement activation.

## Discussion

Healthy host tissues counteract complement-mediated damage by relying on efficient regulators that are either membrane-anchored or circulate as soluble proteins. Constant tick-over activation within the complement cascade marks a potential hazard to any surface; therefore, membrane-bound complement regulators on self-cells ensure that host surfaces are not affected, even after initial opsonization. Membrane-bound complement regulators are embedded in the cell membrane and their regulatory functions are restricted to the host surfaces they protect. Of the four members that are tethered to the plasma membrane, CD35 (CR1) and CD46 (membrane cofactor protein, MCP) are embedded via transmembrane domains, whereas CD55 (decay accelerating factor, DAF) and CD59 (protectin) are fixed to the outside membrane leaflet via a glycosyl phosphatidylinositol (GPI) anchor [33].

CD35 is widely localized on human cells, especially B cells and red blood cells (RBCs) and acts as a cofactor for the inactivation of both C3b and C4b via Factor I (FI) [33, 34]. As shown here, it is not localized in the PNS (Additional file 7: Fig. S7 B-d). CD46 is widely localized on most cell surfaces, with the notable exception of erythrocytes, and protects cells by acting as a cofactor for the FI-mediated degradation of both C3b and C4b [35]. Here we were able to show that CD46 is not localized in compact myelin (Additional file 7: Fig. S7 B-c) and therefore has no role in myelin protection from complement attack. In mice, it is localized in the paranodal area (Additional file 7: Fig. S7 A-c), which is in line with its role in cell signaling [36, 37] and metabolism, as well as development of the neuronal retina, retinal pigment epithelium (RPE), and choroid in C57BL/6 mice. It has an important role in the development of retinal macular degeneration [37, 38].

Decay-accelerating factor (DAF, CD55) is localized on almost all peripheral blood cells as well as endothelial and epithelial cells and has wide localization in multiple tissues. It prevents the formation of new C3 and C5 convertases and accelerates their decay [39, 40]. Recently we have described patients who carry nonfunctional CD55, which led to protein-losing enteropathy [31, 41]. Here we were able to show that CD59, which inhibits the final and most important step of MAC formation, is present in the myelin membrane of both human (Additional file 7: Fig. S7 B-a) and murine (Additional file 7: Fig. S7 A-a) PNS, together with CD55 in humans (Additional file 7: Fig. S7 B-b) and Crry in mice (Additional file 7: Fig. S7 A-d). However, only CD59-deficient patients, and not CD55-deficient patients, are under frequent PNS attack leading to recurrent PNS demyelination episodes and conduction block [3, 4], indicating the crucial role of CD59 in protecting myelin from complement attack.

In this study we show that CD59 is found in compact myelin and in endothelial cells in human peripheral nerve cells. Furthermore, we clearly show the absence of CD59 localization from the node of Ranvier, the site at which nerve impulses propagate [42]. This was in agreement with a finding of Koski et al. [43], who investigated the expression of CD55, CD46, and CD59 on Schwann cells cultured from human sural nerve and myelin membranes prepared from human cauda equina and spinal cord, and found that only CD59 was found on myelin. It is also consistent with findings of Sawant-Mane et al. [44], who showed myelin susceptibility in rats in correlation to the expression of CD59.

Erne et al. [45] demonstrated that rafts found in adult human and murine peripheral compact myelin membranes contain myelin and lymphocyte protein (MAL), the GPI-anchored protein CD59, and substantial amounts of the peripheral myelin protein-22 (PMP22) and P0. Colocalization studies show that CD59 and MAL have an almost identical localization pattern within compact myelin where detergent-insoluble glycolipid-enriched complexes are also found. Our findings agree with Erne et al. [45], who localized CD59 in compact myelin, as shown by us in cross sections, teased fibers, and DRG cultures. In addition, we were able to clearly demonstrate here that neither CD59, CD55, or CD46 are localized in the nodes of Ranvier, as verified in teased fibers and DRGs. In blood vessels, on the other hand, all three membrane-bound regulators, CD59, CD55, and CD46, were found to be localized in the endothelium in the PNS.

The association of CD59 deficiency with demyelination was previously suggested in CD59a KO mice, where induction of acute experimental allergic encephalomyelitis resulted in increased demyelination compared to WT mice [46]. In humans, GBS has been specifically linked to hyperactivity of the complement system by the observed upregulation of terminal complement complex C5b-9 in Schwann cells of the demyelinating fibers. Moreover, deposition of complement components and MAC formation are found in the acute motor neuropathy form of GBS [1, 47–49] and therefore C5b-9 may be found in samples from CD59 deficient patients (to be done in the future if biopsies are available).

In addition, in a murine model of antiganglioside antibody-mediated neuropathy, the passive transfer of anti-GM1 or anti-GD1a antibodies produced a GBS-like syndrome only in the presence of human complement components [50, 51], and eculizumab (which inhibits terminal complement activation) was shown to prevent dysfunction and structural nerve damage [3, 52]. While there are no known autoantibodies in patients with primary CD59 deficiency, nonfunctional CD59 allows spontaneous uncontrolled complement activation that results in severe demyelination and conduction block in the PNS following viral infection [3] and possibly additional triggers.

Along with a reversible conduction block in the hands in these patients, electrophysiological studies of irreversible damage in the foot showed marked delay of distal latencies, low compound muscle action potential amplitudes, and slow conduction velocities (right peroneal and left tibial). The latency of F responses in the left tibial nerve were prolonged, and sympathetic skin responses showed no response. Improvement of the conduction block in distal or intermediate nerve segments was detected following eculizumab treatment [3], suggesting a rapid mechanism of action in the area of the nodes of Ranvier. Thus, some patients with autoimmune (post-infectious antibody-dependent) as well as patients with autoinflammatory (CD59-mutated) GBS have reversible conduction failure attributed to blockade at the nodes of Ranvier without tissue destruction, at least in the early stages of disease progression.

In summary, we were able to show that the nodes of Ranvier have no complement-membrane regulatory proteins CD59, CD46, CD35, or CD55 (or Crry in mice), rendering this area exposed to complement attack. This provides an explanation for observations of complement attack in the nodes of Ranvier in classical autoantibody-dependent GBS [1]. Furthermore, we showed that myelin in the PNS is protected only by CD59 and CD55, but not by CD46 or CD35, rendering it susceptible to complement attack, conduction block, and demyelination in autoinflammatory (non-autoantibody-dependent) situations like CD59 deficiency [3].

### Supplementary Information

**Additional file 1: Figure S1.** Localization of CD59 in murine myelinated cultures. CD59 (green), P0 (red), and Caspr (blue). CD59 is co-localized with P0 and localized in the internodes and paranodes. There is no localization in the nodes of Ranvier. Scale bars = 50 μm.

**Additional file 2: Figure S2.** Electron microscopy (EM) of murine sciatic nerve longitudinal sections. EM pictures of CD59a-deficient murine sciatic nerves. Abnormality in neurofilament orientation is seen. Scale bar = 500 nm.

**Additional file 3: Figure S3.** Localization of complement membrane regulatory CD59 in murine longitudinal sections (A) and a cross section (B) of sciatic nerve. Staining of WT murine sciatic nerve by CD59 (red), CD31 (green), and neurofascin (Nfasc, blue). Section from a 4.5-month-old mouse. Immunolabeling procedure performed with methanol and 0.1% Triton. Scale bars = 50 μm.

**Additional file 4: Figure S4.** Localization of complement membrane regulatory CD55 in murine teased section (A) and longitudinal sections (B) of sciatic nerve. Staining of WT murine sciatic nerve by CD55 (red), CD31 (green), and neurofascin (Nfasc, blue). Sections taken from a 4-month-old mouse. Immunolabeling procedure performed with the TSA method. Scale bars = 20 μm. CD55 staining was localized in Schmidt Lanterman incisure.

**Additional file 5: Figure S5.** Localization of complement membrane regulatory CD46 in murine longitudinal sections (A) and cross section (B) of sciatic nerve. Staining of WT murine sciatic nerve by CD46 (red), CD31 (green), and neurofascin (Nfasc, blue). Section from a 4.5-month-old mouse. Immunolabeling procedure performed with methanol and 0.1% triton. Scale bars = 20 μm.

**Additional file 6: Figure S6.** Localization of complement membrane regulatory Crry in murine teased sections (A) and cross section (B) of sciatic nerve. **A.** Left panels—staining of WT teased fibers. Crry (red), neurofascin (Nfasc, blue), and CD59 (green). Right panels, control using only secondary antibody. **B.** Staining of WT cross section. Crry (red) and CD59 (green). Sections taken from a 4-month-old mouse. Crry staining was localized in the paranodes and the internodes corresponding to compact myelin. Immunolabeling procedure performed with the TSA method. Scale bars = 20 μm.

**Additional file 7: Figure S7.** Differentially localized complement membrane regulatory proteins in peripheral nerve in mice (A) and humans (B). **Mice (A).** CD59 (A-a) was localized along the internodal areas but was absent from the nodes of Ranvier. CD55 (A-b) was localized in the SLI but was absent from the nodes of Ranvier. CD46 (A-c) was localized in the paranodal loops but was absent from the nodes of Ranvier. Crry (A-d) was localized in the paranodal loops and weakly localized in the internodes but was absent from the nodes of Ranvier. Humans (B). CD59 (B-a) and CD55 (B-b) were localized in the area of myelinated nerve fibers. CD46 (B-c) and CD35 (B-d) expressions were absent from the area of myelinated nerve fibers. All regulatory proteins were absent from the nodes of Ranvier. Myelin protein zero (P0), Myelin basic protein (MBP), Ankyrin G (AnkG), NF155 + NF186 (Pan Neurofascin; Nfasc).

## Data Availability

The datasets used during the current study are available from the corresponding author on reasonable request.

## Abbreviations

AnkG: Ankyrin G
BSA: Bovine serum albumin
CR1: Complement receptor 1
Crry: Complement receptor type-1 related gene Y (Crry/p65)
DAB: 3, 3'-Diaminobenzidine
DAF: Decay accelerating factor
DG: Dystroglycan
DRG: Dorsal root ganglia
EM: Electron microscopy
FI: Factor I
GBS: Guillain-Barré syndrome
GD1b: Ganglioside D1b
GM1: Ganglioside-monosialic acid 1
GTC: Gomori-trichrome
GPI: Glycosyl phosphatidylinositol
H&E: Hematoxylin and eosin
HRF20: 20KDa-Homologous restriction factor
HRP: Horseradish peroxidase
IHC: Immunohistochemistry
KO: Knock-out
LFB-PAS: Luxol fast blue-Periodic Acid Schiff
MAC: Membrane attack complex
MAG: Myelin-associated glycoprotein
MAL: Myelin and lymphocyte protein
MBP: Myelin basic protein
MCP: Membrane cofactor protein
mAb: Monocalonal antibody
Necl: Nectin-like (also known as SynCAM or Cadm)
NF: Neurofilament
Nfasc: Pan Neurofascin
NGF: Nerve growth factor
P0: Myelin protein zero
PBS: Phosphate buffer saline
PMP22: Peripheral myelin protein-22
PNH: Paroxysmal nocturnal hemoglobinuria
PNS: Peripheral nervous system
pAb: Polyclonal antibody
RBCs: Red blood cells
PLVAP: Plasmalemma vesicle associated protein
RPE: Retinal pigment epithelium
SLI: Schmidt Lanterman incisures
WT: Wild-type
TB: Toluidine blue
TSA: Tyramide signal amplification
