# Supplementary figures and images for: Complement-membrane regulatory proteins are absent from the nodes of Ranvier in the peripheral nervous system
Source: J Neuroinflammation. 2023 Oct 24;20:245. doi: 10.1186/s12974-023-02920-9 (PMC10594684; doi:10.1186/s12974-023-02920-9)

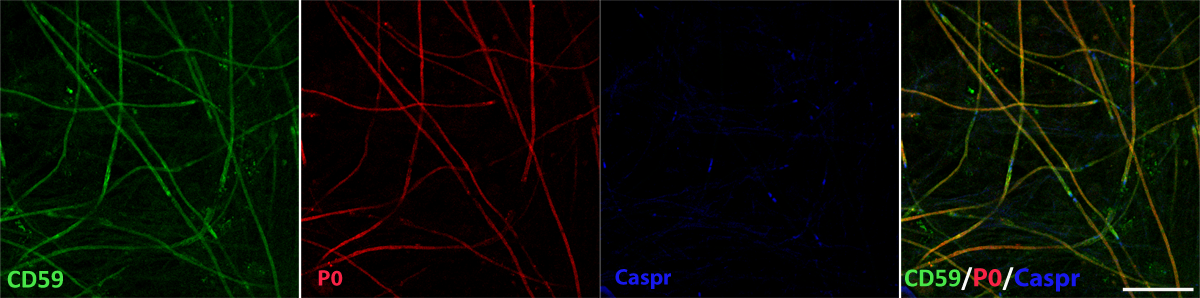

Supplement: Supplementary file 1 — Additional file 1: Figure S1. Localization of CD59 in murine myelinated cultures. CD59 (green), P0 (red), and Caspr (blue). CD59 is co-localized with P0 and localized in the internodes and paranodes. There is no localization in the nodes of Ranvier. Scale bars = 50 μm. [file 12974_2023_2920_MOESM1_ESM.tif]

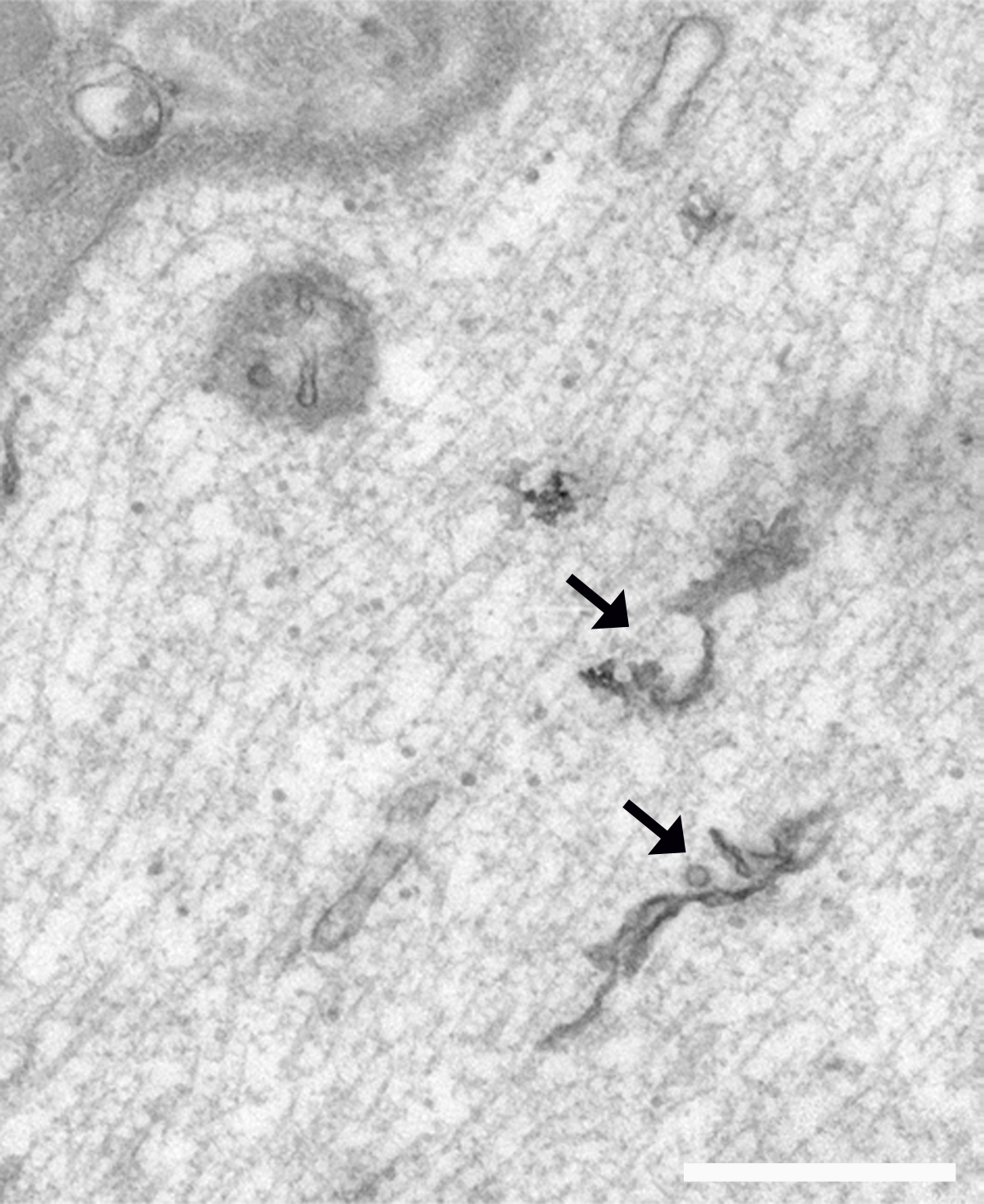

Supplement: Supplementary file 2 — Additional file 2: Figure S2. Electron microscopy (EM) of murine sciatic nerve longitudinal sections. EM pictures of CD59a-deficient murine sciatic nerves. Abnormality in neurofilament orientation is seen. Scale bar = 500 nm. [file 12974_2023_2920_MOESM2_ESM.tif]

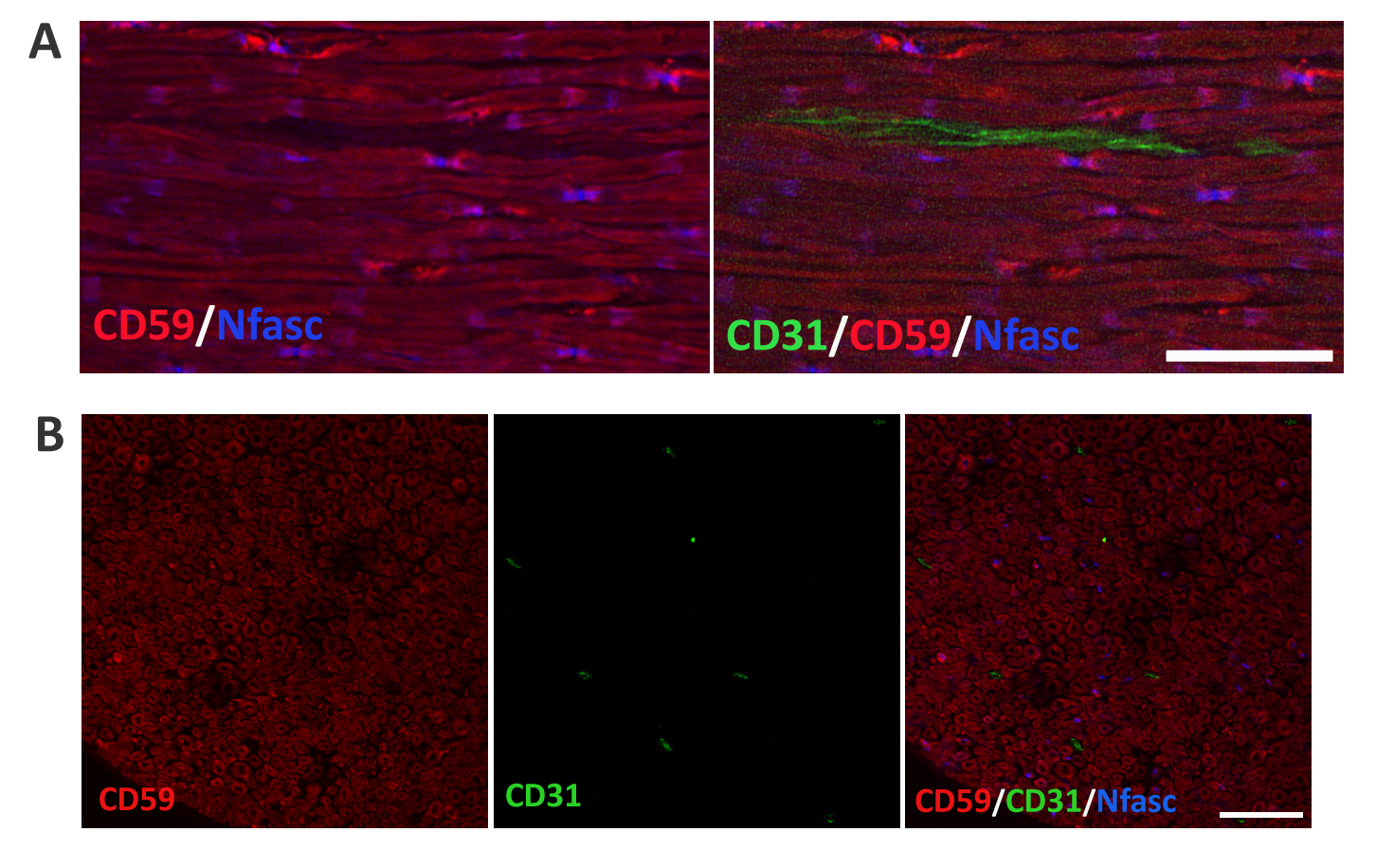

Supplement: Supplementary file 3 — Additional file 3: Figure S3. Localization of complement membrane regulatory CD59 in murine longitudinal sections (A) and a cross section (B) of sciatic nerve. Staining of WT murine sciatic nerve by CD59 (red), CD31 (green), and neurofascin (Nfasc, blue). Section from a 4.5-month-old mouse. Immunolabeling procedure performed with methanol and 0.1% Triton. Scale bars = 50 μm. [file 12974_2023_2920_MOESM3_ESM.tif]

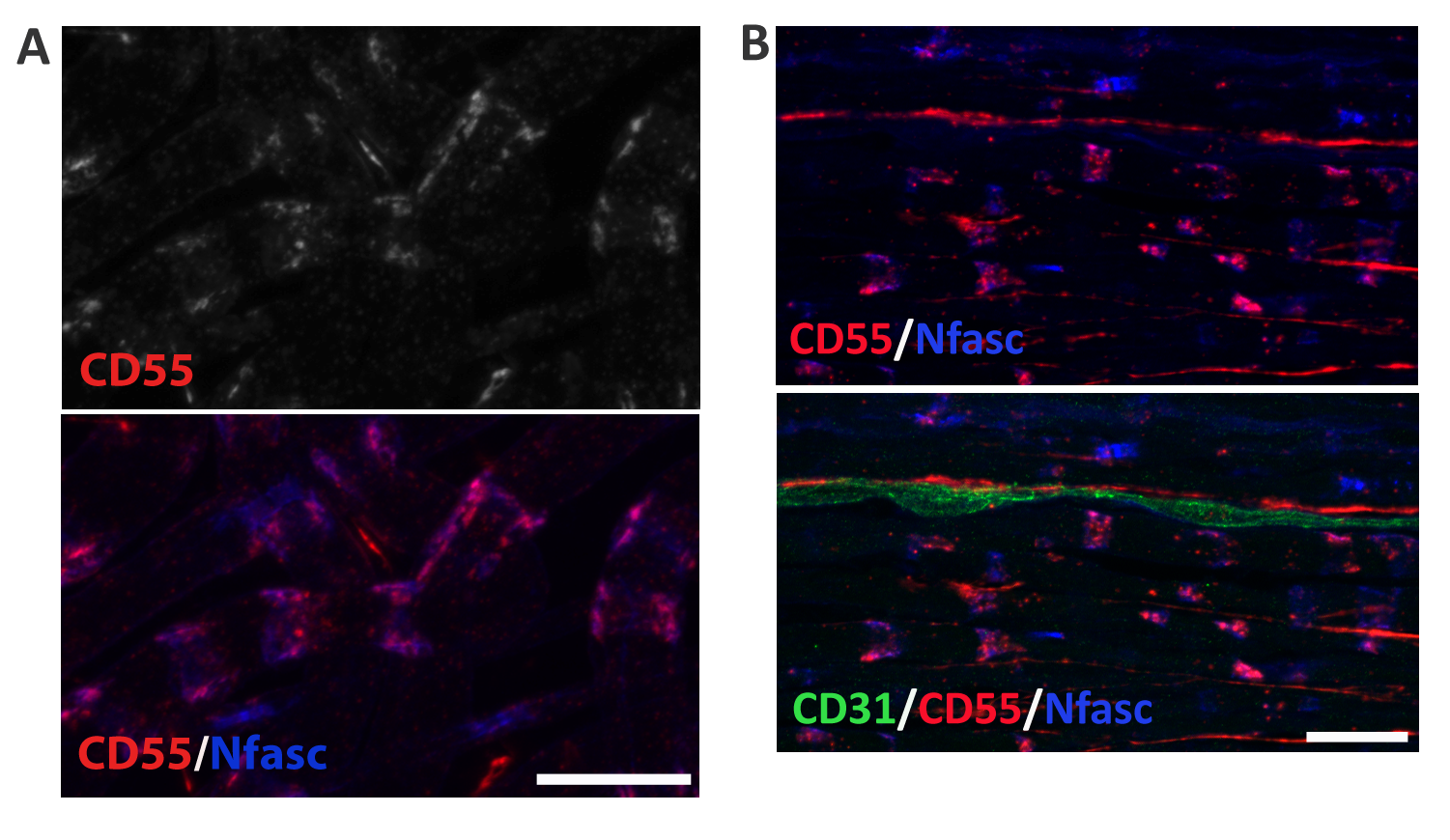

Supplement: Supplementary file 4 — Additional file 4: Figure S4. Localization of complement membrane regulatory CD55 in murine teased section (A) and longitudinal sections (B) of sciatic nerve. Staining of WT murine sciatic nerve by CD55 (red), CD31 (green), and neurofascin (Nfasc, blue). Sections taken from a 4-month-old mouse. Immunolabeling procedure performed with the TSA method. Scale bars = 20 μm. CD55 staining was localized in Schmidt Lanterman incisure. [file 12974_2023_2920_MOESM4_ESM.tif]

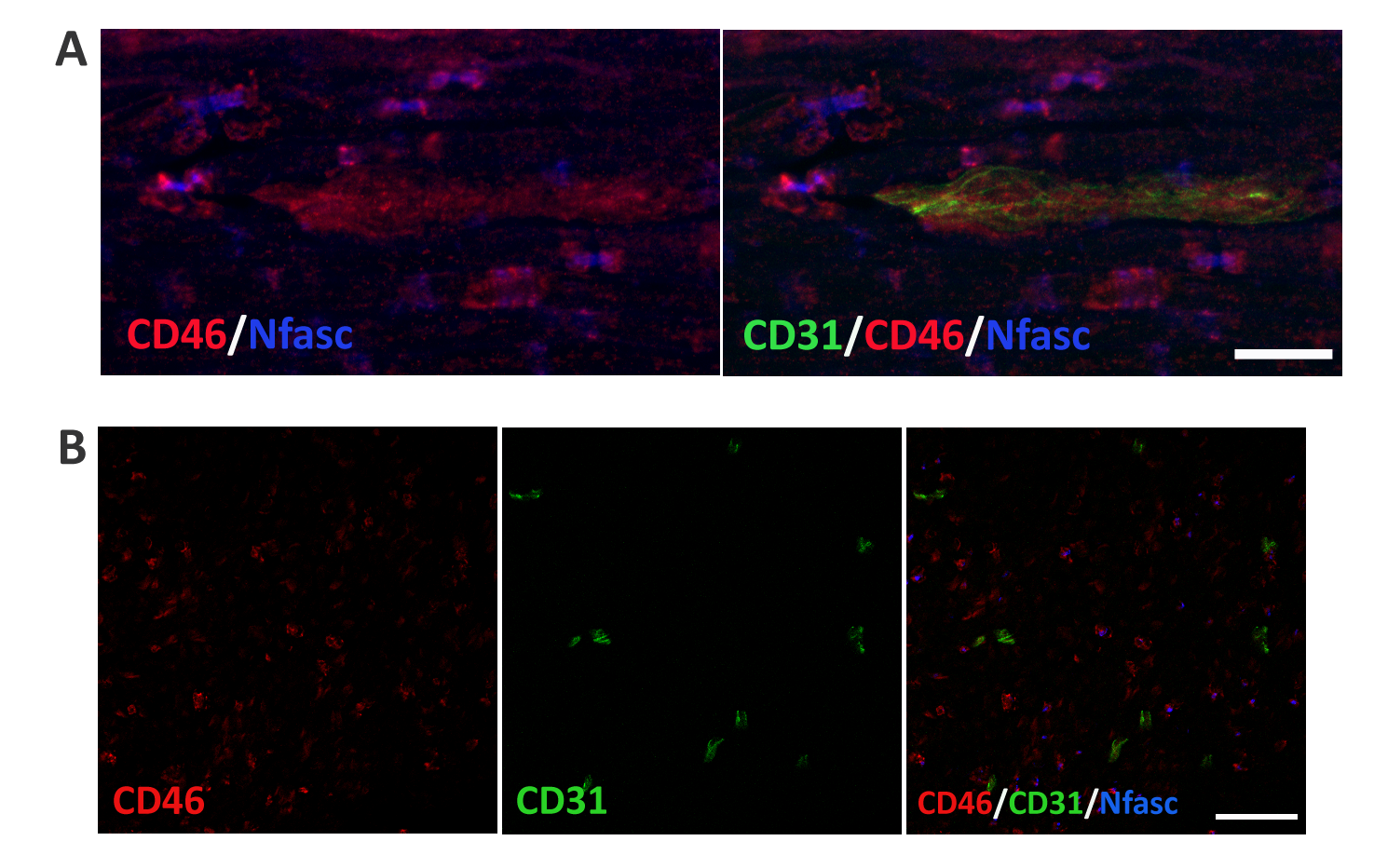

Supplement: Supplementary file 5 — Additional file 5: Figure S5. Localization of complement membrane regulatory CD46 in murine longitudinal sections (A) and cross section (B) of sciatic nerve. Staining of WT murine sciatic nerve by CD46 (red), CD31 (green), and neurofascin (Nfasc, blue). Section from a 4.5-month-old mouse. Immunolabeling procedure performed with methanol and 0.1% triton. Scale bars = 20 μm. [file 12974_2023_2920_MOESM5_ESM.tif]

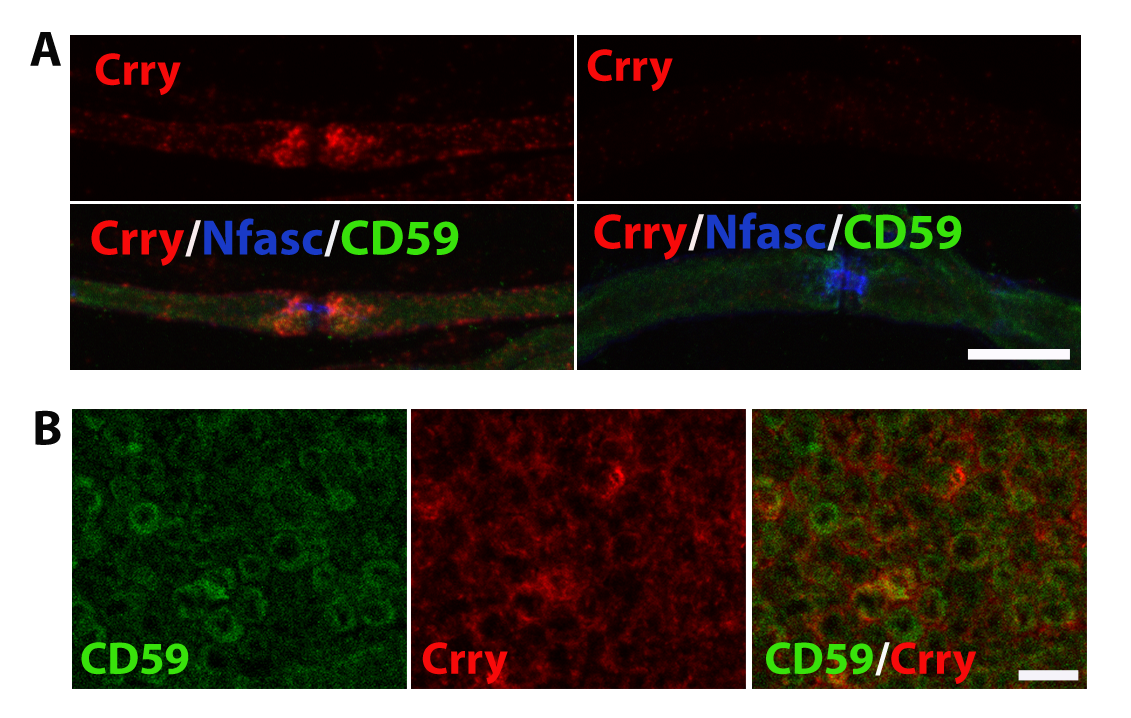

Supplement: Supplementary file 6 — Additional file 6: Figure S6. Localization of complement membrane regulatory Crry in murine teased sections (A) and cross section (B) of sciatic nerve. A. Left panels—staining of WT teased fibers. Crry (red), neurofascin (Nfasc, blue), and CD59 (green). Right panels, control using only secondary antibody. B. Staining of WT cross section. Crry (red) and CD59 (green). Sections taken from a 4-month-old mouse. Crry staining was localized in the paranodes and the internodes corresponding to compact myelin. Immunolabeling procedure performed with the TSA method. Scale bars = 20 μm. [file 12974_2023_2920_MOESM6_ESM.tif]

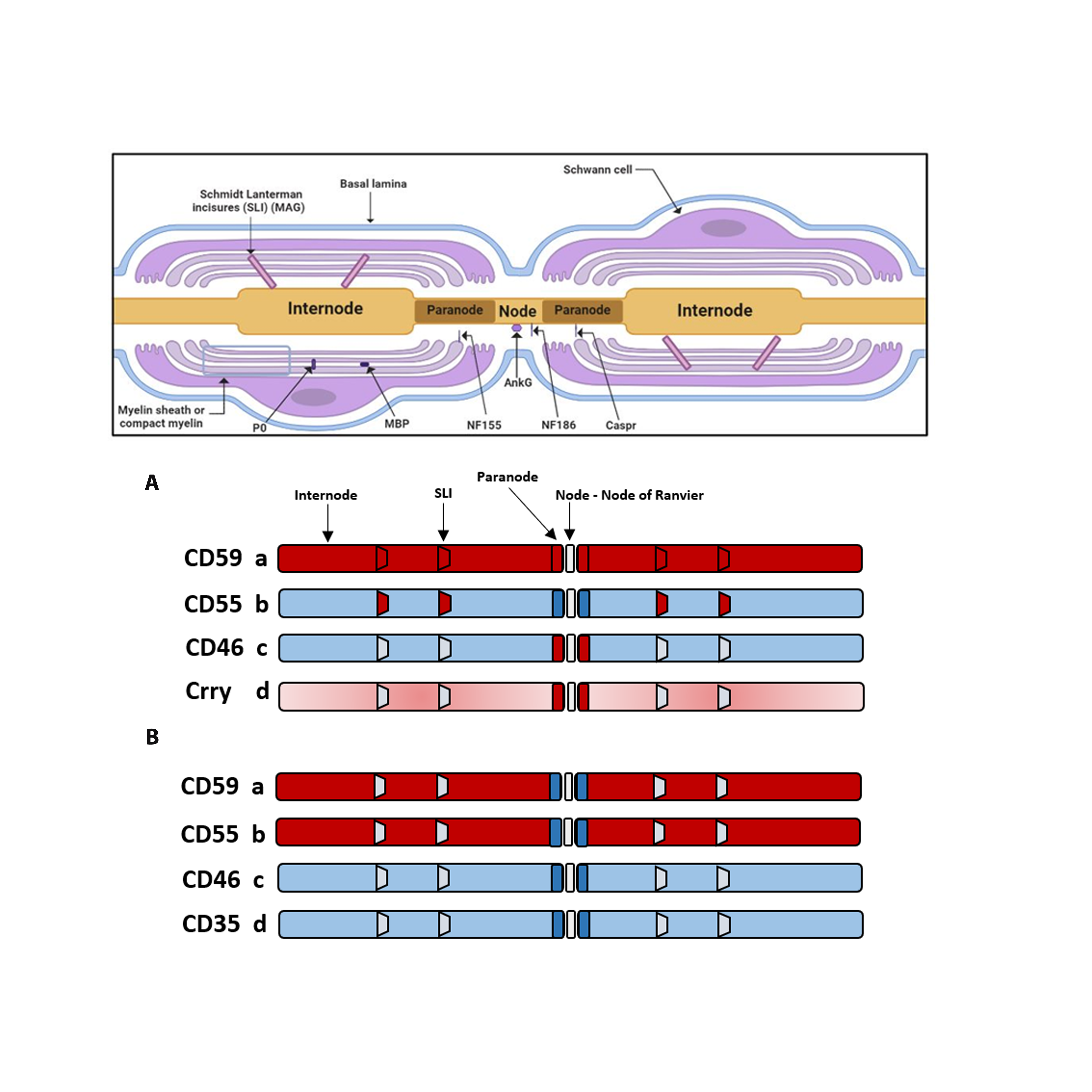

Supplement: Supplementary file 7 — Additional file 7: Figure S7. Differentially localized complement membrane regulatory proteins in peripheral nerve in mice (A) and humans (B). Mice (A). CD59 (A-a) was localized along the internodal areas but was absent from the nodes of Ranvier. CD55 (A-b) was localized in the SLI but was absent from the nodes of Ranvier. CD46 (A-c) was localized in the paranodal loops but was absent from the nodes of Ranvier. Crry (A-d) was localized in the paranodal loops and weakly localized in the internodes but was absent from the nodes of Ranvier. Humans (B). CD59 (B-a) and CD55 (B-b) were localized in the area of myelinated nerve fibers. CD46 (B-c) and CD35 (B-d) expressions were absent from the area of myelinated nerve fibers. All regulatory proteins were absent from the nodes of Ranvier. Myelin protein zero (P0), Myelin basic protein (MBP), Ankyrin G (AnkG), NF155 + NF186 (Pan Neurofascin; Nfasc). [file 12974_2023_2920_MOESM7_ESM.tif]
